# Characterization of genomic regions escaping epigenetic reprogramming in sheep

**DOI:** 10.1093/eep/dvad010

**Published:** 2023-12-20

**Authors:** Camila U Braz, Matilde Maria Passamonti, Hasan Khatib

**Affiliations:** Department of Animal Sciences, University of Illinois Urbana–Champaign, Urbana, IL 61801, USA; Department of Animal Science, Food and Nutrition, Universit’a Cattolica del Sacro Cuore, Piacenza, 29122, Italy; Department of Animal and Dairy Sciences, University of Wisconsin–Madison, Madison, WI 53706, USA

**Keywords:** DNA methylation, epigenetic reprogramming, methionine, nutritional epigenetics, transgenerational epigenetic inheritance

## Abstract

The mammalian genome undergoes two global epigenetic reprogramming events during the establishment of primordial germ cells and in the pre-implantation embryo after fertilization. These events involve the erasure and re-establishment of DNA methylation marks. However, imprinted genes and transposable elements (TEs) maintain their DNA methylation signatures to ensure normal embryonic development and genome stability. Despite extensive research in mice and humans, there is limited knowledge regarding environmentally induced epigenetic marks that escape epigenetic reprogramming in other species. Therefore, the objective of this study was to examine the characteristics and locations of genomic regions that evade epigenetic reprogramming in sheep, as well as to explore the biological functions of the genes within these regions. In a previous study, we identified 107 transgenerationally inherited differentially methylated cytosines (DMCs) in the F1 and F2 generations in response to a paternal methionine–supplemented diet. These DMCs were found in TEs, non-repetitive regions, and imprinted and non-imprinted genes. Our findings suggest that genomic regions, rather than TEs and imprinted genes, have the propensity to escape reprogramming and serve as potential candidates for transgenerational epigenetic inheritance. Notably, 34 transgenerational methylated genes influenced by paternal nutrition escaped reprogramming, impacting growth, development, male fertility, cardiac disorders, and neurodevelopment. Intriguingly, among these genes, 21 have been associated with neural development and brain disorders, such as autism, schizophrenia, bipolar disease, and intellectual disability. This suggests a potential genetic overlap between brain and infertility disorders. Overall, our study supports the concept of transgenerational epigenetic inheritance of environmentally induced marks in mammals.

## Introduction

In differentiated mammalian cells, DNA methylation patterns are relatively stable and heritable. However, the mammalian genome undergoes two global epigenetic reprogramming events that erase and re-establish DNA methylation patterns, first in the primordial germ cells (PGCs) –the precursors of sperm and egg – during gametogenesis and second, in the pre-implantation embryo after fertilization [1]. The reprogramming events in PGCs include global DNA demethylation, erasure of genomic imprints, X-chromosome reactivation, and chromatin reorganization [2]. These epigenetic reprogramming events are essential for generating embryos with broad developmental potential through the differentiation between the trophectoderm and the inner cell mass [3]. Following the first epigenetic reprogramming event, Popp *et al*. [4] estimated that male and female germ cells have ∼60% and 70% less DNA methylation, respectively, than embryonic stem cells. However, despite the global loss of methylation, some imprinted sequences and retrotransposons escape epigenetic reprogramming and maintain their methylation status [1]. Imprinted genes evade the second round of reprogramming and the subsequent wave of *de novo* methylation to maintain their parental origin marks across generations [5]. In addition, it is believed that the retention of DNA methylation in transposable elements (TEs) prevents the reactivation of these elements [3].

Although the purpose of the reprogramming process remains to be elucidated, this mechanism may be required to prevent epigenetic signatures acquired during gametogenesis and early development or imposed by the environment from being transferred to the offspring [6, 7]. Therefore, genomic regions escaping reprogramming may be involved in epigenetic inheritance [1]. Furthermore, errors or environmentally induced modifications that occur during the two waves of erasure and re-establishment of epigenetic marks could induce DNA methylation retention leading to epigenetic inheritance [7]. One of the most studied examples of epigenetic inheritance affected by environmental factors is the IAP (intracisternal A particle) transposon upstream of the agouti locus in mice. The supplementation of methyl donors to pregnant mothers led to DNA methylation changes in the IAP locus, affecting agouti gene expression and leading to the inheritance of coat color and DNA methylation changes by the offspring [8]. The maternal epigenetic inheritance of the agouti phenotype is due to an incomplete erasure of DNA methylation at the IAP locus [9].

There is growing evidence that environmentally altered epigenetic marks that escape reprogramming affect the phenotypes of the next generation. Using mouse models, several environmental toxicants have been shown to induce transgenerational inheritance of altered sperm DNA methylation associated with pathologies and diseases [10]. Zheng *et al*. [11] reported that mice exposed to long-term psychological stress showed sperm epigenetic alterations and increased health risks across generations. However, the fact that epigenetic marks that escape reprogramming may reflect the parents’ experience suggests that those marks may be involved in preparing the offspring to cope with environmental challenges, that is, having the potential to be adaptive with implications for heredity, breeding, and evolution [6, 7].

In a previous study, we showed that paternal exposure to a methyl donor diet induced transgenerational epigenetic inheritance (TEI) of DNA methylation changes in sperm that passed to subsequent generations [12]. These DNA methylation changes were associated with growth and fertility traits in sheep across generations [12]. Interestingly, many TEI marks were in genomic regions other than imprinted genes and TEs known to resist epigenetic reprogramming, indicating that other genomic regions could be subject to incomplete reprogramming. Other studies in mice and humans have also identified genomic sequences that resist the erasure of DNA methylation during the two waves of epigenomic reprogramming [13, 14]. However, there is limited knowledge about environmentally altered TEI marks that escape epigenetic reprogramming, particularly in species other than mice and humans. Therefore, this study aims to characterize the types and locations of genomic regions escaping epigenetic reprogramming in sheep and discuss the biological functions of genes in these regions. Elucidating the functions of these genes is important because it could improve our understanding of the mechanisms of TEI and contribute to the discussion about the inheritance of acquired traits in mammals.

## Results and discussion

During adult gametogenesis in males, a genome-wide remodeling of the epigenome occurs, where sperm DNA is demethylated and re-methylated, and chromatin is reorganized by replacing most of the histones with protamines to permit supercoiling and compaction [15]. During this period of nuclear remodeling, the sperm epigenome is transiently vulnerable to environmental influences, leading to epigenetic shifts [16]. These environmentally induced germline changes can be passed down to subsequent generations – known as TEI – due to an incomplete erasure of epigenetic marks during the reprogramming process [7]. In a previous study, we exposed F0 rams to a methionine–supplemented diet during gametogenesis (from weaning to puberty) and identified 107 DMCs that were maintained in the F1 and F2 generations in a TEI manner [12]. The results suggest that these environmentally induced TEI sites may escape epigenetic reprogramming. Out of the 107 DMCs transgenerationally inherited (TEI DMC), 82, 20, and 5 were found in CG, CHH, and CHG context, respectively (Table 1). The characterization of genomic regions and the functions of genes escaping epigenetic reprogramming are discussed here.

**Table 1:** Types of inheritance of DMCs across F0, F1, and F2 generations after exposure of F0 males to a methionine–supplemented diet

	DMCs			DMCs in genes			DMCs in TEs		
Type of inheritance^a^	CG	CHH	CHG	CG	CHH	CHG	CG	CHH	CHG
F0–F1–F2	82	20	5	28	8	1	49	14	5
F1–F2	143	39	8	39	21	7	92	29	7
F0–F2	238	74	23	73	27	7	164	49	14

a DMCs between methionine-treated and control groups detected in all generations (F0–F1–F2); only in F1 and F2, not in F0 (F1–F2); or those detected in F0 and F2, skipping F1 (F0–F2).

### Genomic locations of TEI DMCs

**Figure 1:**
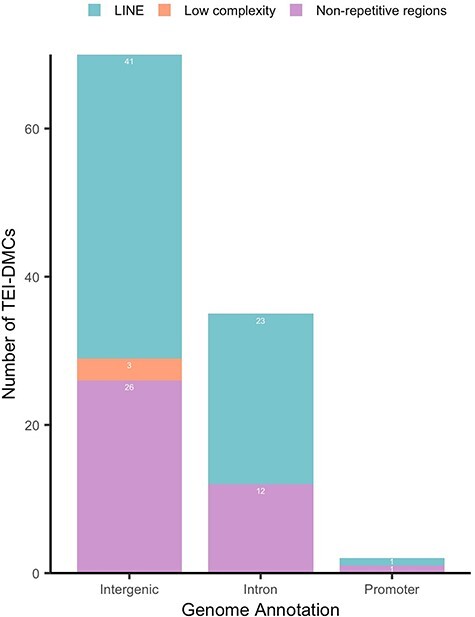
Genome annotation of the transgenerational epigenetic inherited differentially methylated cytosines (TEI DMCs) found in F0, F1, and F2 generations in sheep. Genomic locations are displayed related to the numbers of TEI DMCs located in each class of repetitive elements (LINE, low complexity, or non-repetitive regions) for each genomic region. LINE = long interspersed nuclear element

The identified 107 TEI DMC reside in intergenic (65%), intronic (33%), and promoter regions (2%) (Fig. 1). These results are consistent with our previous study using reduced representation bisulfite sequencing, in which we detected 48.7% of the DMCs mapped to intergenic regions, 40% in intronic regions, 8% in promoter regions and exons, while <5% mapped to 3ʹ and 5ʹ UTRs [17]. Many DMCs were related to developmental processes, anatomical structure development, nervous system development, and growth.

While it is well-established that DNA methylation in the promoter can be negatively associated with gene expression, the methylation status in the gene body can show a positive correlation [18]. DNA methylation in intergenic regions may regulate microRNA expression and contribute to genomic stability and conservation [19]. The retention of methylation marks in these regions is not as well documented as for imprinted and TEs. The prominent hypotheses are related to a specific DNA binding factor, histone mark enrichment, or positional context, including regions close to telomeres or flanking TEs [2, 13]. Studies have found epigenetic marks escaping reprogramming located in subtelomeric regions and pericentromeric repeats [13].

A large portion of the TEI DMCs (68 TEI DMC, 63.5%, Table 1) were found to be located within repetitive element (RE) regions, with the majority within long interspersed nuclear elements (LINE) and few in low complexity regions (Fig. 1). Low-complexity repeats are regions containing simple sequence repeats that are possibly involved in chromatin accessibility, dictating the timing and level of gene expression, and may play a function in early embryonic development [20]. TEs are a family of DNA sequences that can copy themselves and move to different positions in the genome, and they constitute up to two-thirds of the mammalian genome [21]. The host genome typically silences these elements with epigenetic marks, such as DNA methylation, to prevent their replication and insertion into functional genomic regions, which can destabilize the genome and perturb the cellular transcriptome [22].

Our results suggest that the methionine–supplemented diet altered the DNA methylation levels of many TEs, and those modifications were passed down to subsequent generations. Previous studies have reported similar findings regarding the dietary sensitivity of TE alteration. A methyl-rich diet provided to agouti viable yellow (*A^vy^*) female mice during pregnancy, modulated the methylation status at the TE close to the *A^vy^* gene, driving its expression and leading to yellow coat color and obesity [23]. The hypomethylated status, yellow coat color, and obesity were inherited over two generations through the maternal line [24]. Another example of transmitted epialleles harbored by TE is the murine Axin fused (*Axin^Fu^*) allele. The study showed that differential DNA methylation at a TE within *Axin^Fu^* is correlated with the kinked tail phenotype and can be transmitted through the maternal and paternal lines [25]. In a study where female mice received methyl donor supplementation before and during gestation, the offspring showed increased DNA methylation at *Axin^Fu^* and reduced incidence of tail kinking [26]. While these studies showed intergenerational epigenetic inheritance of environmentally altered TEs, to our knowledge, our study reports the first evidence of TEI of TEs mediated by paternal diet. Indeed, Guo *et al*. showed that most of the TEs’ DNA methylation patterns may not be reprogrammed in human PGCs, thus maintaining their methylation status, which has also been noted in the mouse germline, allowing for the transmission of their DNA methylation pattern to the next generation [27]. In contrast, Zheng *et al*. [11] observed that only 2–5% of TEs escaped the first round of demethylation in mice. Future studies should further explore mechanisms underlying DNA methylation within TEs escaping reprogramming.

Of the 68 TEI DMCs located in TEs (Table 1), 29 (∼43%) were hypermethylated, with methylation differences varying from 20.2% to 49.1% in the methionine-treated group compared to the control group (Supplementary Table S1). The 39 hypomethylated TEI DMC in TEs (∼57%) showed methylation differences varying from −21.1% to −57.5% in the methionine-treated group (Supplementary Table S1). The TEs’ hypermethylation could be related to their transcriptional repression [28]. Conversely, hypomethylation of TEs has been shown to promote their reactivation and transposition in the genome, and it has been associated with cancer, neurodevelopmental diseases, and infertility in humans [29, 30]. On the other hand, TE reactivation could serve as a variability source, inducing an adaptive phenotypic variation in response to the environment [31]. Notably, 95% of TEI DMCs residing in TEs are in LINEs, with the majority being active elements LINE-1 (L1) (Supplementary Table S1). L1 is the most abundant mammalian TE, comprising nearly 20% of the mammalian genome [32], and L1 hypomethylation has been observed in various cancer cells [33].

We identified a TEI DMC (chromosome 1 at 45 919 820 bp) within a TE upstream (9679 bp) of the imprinted gene *DIRAS3* gene. The expression of imprinted genes is tightly regulated, and slight changes in their expression can lead to significant changes in phenotype [34]. Our results suggest a disturbance of the TE upstream of *DIRAS3* by the methionine–supplemented diet, where methionine-treated animals had a hypomethylated TEI DMC in the exposed and the two subsequent generations (−35.7% in F0, −25.4% in F1, −30.7% in F2). This epigenetic modification can potentially alter *DIRAS3* expression status since evidence that the epigenetic state of TE can persist transgenerationally and impact the expression of neighboring genes has been shown [24]. We observed that, on an average, the methionine-treated group with hypomethylated TEI DMC near *DIRAS3* also exhibited lower fertility status, having smaller scrotal circumference when compared to the control group [12]. Likewise, low-level methylation at *DIRAS3* has been previously related to male infertility in humans [35]. Future studies should investigate the impact of hypomethylation in CG sites near *DIRAS3*.

Although most TEI DMCs detected were in repetitive element regions, 13 TEI DMCs were found in non-repetitive regions (Fig. 1). DMCs in non-repetitive regions have previously been reported [15], suggesting these regions are also susceptible to environmental epigenetic modifications and may escape epigenetic reprogramming. Guibert *et al*. [13] identified single-copy sequences and LTR retroelements that escape epigenetic reprogramming in both PGCs and preimplantation embryos in mice, suggesting potential TEI. Notably, some of these sequences were not near repetitive elements, a finding consistent with our results in sheep. DNA methylation analysis of human PGCs revealed many genomic regions escaping epigenetic reprogramming [14]. Interestingly, many of these escapee regions were predominantly depleted of retrotransposons or located at least 1 kb away from any repeat family [14]. Thus, mouse and human studies support our hypothesis that genomic regions escaping epigenetic reprogramming are candidates for TEI.

### Genomic regions escaping epigenetic reprogramming and skipping generations

The occurrence of TEI requires that phenotypic and epigenetic effects must be observed in subsequent generations in the absence of exposure to the inducing agent or environmental factor that initiated the change [7, 36]. Therefore, we speculate whether DMCs detected in F1 and F2 (not in F0) or those detected in F0, not in F1, and found again in F2 should be considered TEI DMCs. Here we will denote such DMCs as F1–F2 DMCs and F0-F2 DMCs. We found 143, 39, and 8 F1-F2 DMCs, and 238, 74, and 23 F0–F2 DMCs in CG, CHH, and CHG contexts, respectively (Table 1, Supplementary Table S1). A possible explanation for common DMCs found only between F1 and F2 is that the methyl donor induced epigenetic marks in the germ cells (F1) but not in the F0 generation, which were transmitted to the F2. Another explanation is that the methyl group was added to the cytosines of the F0 after the F0 semen was collected, resulting in a carry-over effect. Also, the differential methylation could appear after the post fertilization reprogramming.

Epimutations skipping generations have been reported previously [10, 37]. A study of gestating female rats exposed to vehicle control or atrazine found common epimutations between F1 and F2 but not in the exposed generation [37]. Kubsad *et al*. [10] exposed female rats (F0 generation) to glyphosate during 8–14 days of gestation and investigated the transgenerational effects on pathology and disease in the offspring. No significant pathology was observed in the first two generations (F0 and F1) of animals exposed to glyphosate. However, a significant increase in pathology (e.g. prostate disease frequency and male obesity) was observed in the F2 and F3 generations of animals [38]. This suggests that glyphosate can cause epigenetic transgenerational inheritance of disease. These results corroborate our previous findings that inherited epimutations that skip generations may be widespread, although the mechanisms underlying these observations are poorly understood and warrant further investigations.

### Functions of genes escaping epigenetic reprogramming

Recently, we showed that the sperm epigenome is sensitive to environmental insults – such as nutrition – that affect embryo gene expression [17] and subsequent generations [12, 39]. We showed that the F0 methionine–supplemented diet affected body weight in F2 females, loin muscle depth (a growth-related trait) in F2 males, and scrotal circumference (associated with male fertility) in both F1 and F2 males [12]. The TEI genes affected by paternal nutrition and escaping epigenetic reprogramming have been associated with growth and development, male fertility, cardiac disorders, and neurodevelopment. Table 2 describes the possible functions of these genes.

**Table 2:** Functions of genes escaping epigenetic reprogramming in growth, fertility, cardiac disorders, and neurodevelopment

Gene ID	Function	Reference
ADGRV1	Associated with several forms of epilepsy	[100]
ASTN2	Codes for a membrane protein implicated in synaptic function; associated with intellectual disability, attention-defict hyperactivity disorder, Alzheimer’s disease, bipolar disorder, major depressive disorder, response to anti-psychotic treatment, anhedonia, neuroticism, and mood instability	[52, 60, 101–103]
	Associated with cardiometabolic traits such as blood pressure and obesity	[104]
AUTS2	A crucial gene associated with a wide range of neuropsychological disorders, including autism spectrum disorder, intellectual disability, schizophrenia, and epilepsy	[105]
AXDND1	Required for spermatid differentiation; associated with male sterility	[83]
BRINP3	Involved in anxiety response and sociability in mice	[106]
CAP2	Controls dendritic spine morphology and synaptic plasticity	[107]
	Associated with body height in sheep	[108]
CATSPER3	Crucial for sperm motility, associated with male fertility	[109]
CDH12	Affects axon extension; associated with Bipolar Disease, schizophrenia, and dependency from methamphetamine and alcohol	[110]
	Associated with bone growth in chickens	[111]
CFAP299	Involved in spermatogenesis, a candidate cause of male infertility	[112]
CNTN5	Involved in the development of the cerebral cortex; associated with attention-deficit hyperactivity disorder, anorexia nervosa, and substance abuse	[47, 113–115]
CNTNAP2	Associated with autism spectrum disorders and epilepsy	[50, 116, 117]
COL19A1	Involved in the formation of hippocampal synapses and perineuronal nets	[58]
	Encodes a type XIX collagen and regulates muscle development	[38]
	Regulates cardiac extracellular matrix structure and ventricular function in mice	[40]
CSGALNACT1	Required for normal cartilage development; associated with mild skeletal dysplasia	[118]
	Involved in neuronal regeneration and plasticity; knockdown alters social behavior in mice	[119, 120]
CTNNA3	Associated with late-onset Alzheimer’s disease, autism spectrum disorder susceptibility, and schizophrenia	[48, 49, 121]
	DMC within this gene found to be associated with male fertility	[68]
	Involved in dilated cardiomyopathy-associated phosphorylation	[122]
	Associated with weight, height, body length, and chest circumference in Hu sheep	[108]
DIRAS3	Its DMR considered a marker of sperm DNA damage, thus associated with idiopathic male infertility	[35]
	Regulates growth, development, and adipogenesis.	[41]
DLG2	Associated with intellectual disability, behavioral disorders, and Parkinson’s disease	[57, 123]
	Associated with testis development and male fertility in mice	[124]
IL1RAP	Involved in neuronal synapse and neuronal development; associated with Alzheimer’s disease and schizophrenia	[53, 125, 126]
LOC105602588	Uncharacterized	
LOC101123029	Uncharacterized	
LOC105613000	Uncharacterized	
LPAR1	Essential for maintaining the normal functions of the central nervous system; alterations in function or expression associated with neurodevelopmental and neuropsychiatric disorders and brain cancer	[61]
	Associated with semen quality in Holstein–Freisian bulls	[63]
LRRIQ3	Associated with schizophrenia, migraine, and severe intellectual disability	[54, 127, 128]
	Associated with muscle development in swine	[129]
NOD1	Plays a critical role in the control of host defense and inflammation in particular during intracerebral hemorrhage	[130, 131]
SCLT1	Ciliary gene whose mutations are associated with ciliopathy phenotypes (as oro-facio-digital syndrome type IX, Senior–Loken syndrome, Bardet–Biedl syndrome); associated with alcohol use behavior in trauma-exposed human populations	[132, 133]
SEC23IP	Associated with intellectual disability, craniofacial and brain malformations, and attention-deficit/hyperactivity disorder	[51, 128]
	Required for spermiogenesis	[45]
STAG1	Cohesin subunit participating in sister chromatid cohesion and 3D genome organization; associated with neurodevelopmental disorders and syndromic intellectual disability	[55, 134, 135]
STK32B	Associated with anxiety disorders in adolescents and with essential tremor	[136, 137]
	Associated with sperm concentration in bovine	[138]
SVEP1	Associated with coronary artery disease	[139, 140]
THOC1	Involved in presynaptic development and plays roles in dopamine neuron survival, associated with herding behavior (fear) in dogs	[64, 65]
	Required for testis and embryo development in mice	[66, 141]
TLL1	Under stress conditions, indirectly affects neurogenesis; proposed as a susceptibility gene for post-traumatic stress disorder	[142, 143]
TLN2	Associated with depression and Alzheimer’s disease	[142, 143]
	Associated with cardiomyopathy	[144]
TMEM245	Associated with schizophrenia (often found as C9orf5)	[48]
TNNI3K	Implicated in several cardiac phenotypes and diseases, such as cardiomyopathy; deletion reduces infarct size and cardiomyocyte death	[145]
ZBTB20	Involved in astrocytogenesis during mammalian neocortical development; hypermethylation associated with major depressive disorder	[56, 146]
	Associated with cryptorchidism	[147]

Genes harboring TEI DMCs that play roles in growth traits were *DIRAS3, CTNNA3, CAP2, COL19A1, LRRIQ*3, and *CDH12*. The genes *ASTN2, COL19A1, TLN2, SVEP1*, and *TNNI3K* were found to be involved in heart diseases and cardiometabolic functions. Some genes (e.g. *DIRAS3, CTNNA3, LRR1Q3*, and *COL19A1*) have multiple functions related to the four categories (fertility, neurodevelopment, growth, and cardiac functions) identified in this study. For example, *COL19A1*, which encodes a type XIX collagen and regulates muscle development [38], is also involved in regulating ventricular functions in the mouse [40]. *DIRAS3* has been reported to regulate growth, development, and adipogenesis [41] and infertility in men [42]. Although the mechanisms by which DNA methylation signatures acquired in the sperm through environmental exposures affect adult traits remain elusive, there is strong evidence of this link. Aissa *et al*. [43] reported that a methionine–supplemented diet affected cardiovascular disease-related genes in mice. Also, Morgan *et al*. [44] showed an association between paternal diet and offspring vascular homeostasis and concluded that sperm and seminal plasma can influence cardiovascular health.

Among the genes escaping epigenetic programming identified in this study, those related to spermatogenesis and male fertility include *DIRAS3, CTNNA3, DLG2, LPAR1, AXDND1, YBX3, THOC1, GK2, CATSPER3, ZBTB20, CFAP299*, and *SEC23IP* (Table 2). *DIRAS3* is an imprinted gene with monoallelic paternal expression and has various known functions related to tumor suppression; downregulation of the PI3K/AKT, JAK/STAT, and RAS/ERK signaling pathways; autophagy induction; and inhibition of adipogenesis [35]. Altered DNA methylation in *DIRAS3* has been associated with differential levels of sperm DNA fragmentation in men [38]. In addition, low methylation levels of *DIRAS3* have been associated with infertility in men [32]. *SEC23IP* has been related to defects in spermiogenesis [45]. Also, Borgel *et al*. [46] reported that nonimprinted genes escaping epigenetic reprogramming were active in gamete production in the male germline. These genes resist global DNA methylation reprogramming during preimplantation development by inheriting promoter DNA methylation from parental gametes. The authors concluded that transgenerational transmission of DNA methylation can occur in a substantial proportion of the mouse genome [46]. These results support our findings in sheep that some nonimprinted genes, influenced by the paternal diet, can escape epigenetic reprogramming, and affect male fertility and embryo development.

Of the 34 genes identified here with TEI DMC in sperm, 21 genes have been reported to be involved in neural development and brain disorders (Table 2). *ASTN2, CNTNAP2, STAG1, LRRIQ3, TMEM245, IL1RAP, CTNNA3, CDH12, BRINP3, CNTN5*, and *SEC23IP* have been associated with neurodevelopmental disorders, such as autism, schizophrenia, attention-deficit/hyperactivity disorder, bipolar disease, intellectual disability, and global developmental delay [47–55]. Hypermethylation in the coding region of *ZBTB20* was found to be associated with major depressive disorder [56], and single nucleotide polymorphisms (SNPs) in *DLG2* were found to be associated with Parkinson’s disease [57]. Central neurons express COL19A1, which is necessary for the formation of hippocampal synapses [58] and may contribute to complex brain disorders. Interestingly, *COL19A1* is also involved in the regulation of muscle growth and ventricular functions (Table 2). Genes escaping epigenetic reprogramming and expressed in the brain were also found in the study of Tang *et al*. [14]. DNA methylation profiling of human PGCs revealed that many repeat-poor sequences (<10% overlap with repeats) escaping global DNA erasure were frequently expressed in the brain and play roles in neural development [14]. Also, these genes were associated with obesity traits, schizophrenia, and multiple sclerosis. Consistent with our results, the authors found that conserved genes escaping epigenetic reprogramming in humans and mice had predominantly brain- and growth-related functions [14]. A different study found that a paternal methyl-donor-rich diet supplemented 6 weeks before mating affected hippocampus-dependent learning and memory tasks in the F1 generation but not in the F2 generation [59]. Interestingly, these behavioral changes were associated with gene expression and promoter methylation changes around the transcription start site of *Kcnmb2* in the F1 generation. The authors speculated that paternal diet could affect mental health in the offspring. When analyzing all common genes harboring F1–F2, and F0–F2 DMCs, we observed 10 significant biological processes [false discovery rate (FDR) < 0.05], with the majority related to nervous system functions (Table 3, Supplementary Table S2), similar to the roles played by several TEI genes.

**Table 3:** Biological processes enriched by genes commonly detected with DMCs in F1 and F2 generations or in F0 and F2 generations

Term name	Term id	FDR	Genes
Head development	GO:0060322	0.0099	13
Brain development	GO:0007420	0.0109	12
Regulation of anatomic structure morphogenesis	GO:0022603	0.0193	13
Cell adhesion	GO:0007155	0.0225	16
Nervous system development	GO:0007399	0.0225	21
Anatomical structure morphogenesis	GO:0009653	0.0225	25
Developmental process	GO:0032502	0.0225	42
Anatomic structure development	GO:0048856	0.0225	40
Cell part morphogenesis	GO:0032990	0.0350	10
Multicellular organismal process	GO:0032501	0.0380	45

Interestingly, many TEI genes were found to have roles in both spermatogenesis and neurodevelopment (Table 2). For example, lysophosphatidic acid receptor 1 (*LPAR1*) was highly methylated in the sperm of methionine–supplemented rams and the F1 and F2 generations compared to controls [12]. LPAR1 signaling can inhibit programmed cell death and enhance neurogenesis through premature cell cycle exit and neuronal differentiation [60]. *LPAR1* deletion is associated with neurodevelopmental disorders in humans and mice [61]. Furthermore, knockout of the *LPAR1* gene in mice resulted in azoospermia and age-related progression of germ cell degeneration [62], and a splicing mutation in *LPAR1* was found to be significantly associated with semen quality in bulls [63]. The THO complex 1 (*THOC1*) gene (Table 2) is a part of the transcription/export complex that functions in the maturation and export of mRNA from the nucleus to the cytoplasm. The *THOC1* gene is involved in both brain and testis functions. Maeder *et al*. [64] reported that *THOC1* is involved in dopamine signaling and survival and is highly conserved from yeast to mammals. SNPs in *THOC1* were found to be associated with dog behavior traits such as herding, predation, and temperament [65]. Wang *et al*. [66] found that a deficiency of *Thoc1* in mice led to abnormal testis development. Another example of sperm-identified genes with brain function found in our study is *CTNNA3* (Table 2). *CTNNA3* has been found to be downregulated in chicken testis under heat stress conditions, suggesting a role in spermatogenesis [67]. In addition, *CTNNA3* is imprinted in humans, and a DMC within the gene has been associated with loss of fertility in men [68]. *CTNNA3* is associated with several brain disorders, such as autism [69] and Alzheimer’s disease [70]. Also, in a previous study, we investigated the effects of paternal methionine supplementation on sperm DNA methylation and the embryo transcriptome in sheep and found DMCs in sperm-active genes that also participate in nervous system development [17].

These results suggest that the same set of genes could influence both brain and infertility disorders. Indeed, there is accumulating evidence of several shared features between the brain and testis, including gene expression profiles, membrane receptors, calcium signaling, fatty acid levels, and others [71]. Gene expression analysis in 15 human and 15 mouse tissues revealed that the brain and testis share similar gene expression profiles more than any other pairs of tissues [72]. A comparison of the brain and testis proteomes with 31 other tissues revealed that the human brain and testis shared the greatest similarity in protein expression [71]. Twelve of the 34 TEI genes found in our study are among the shared genes reported by Matos *et al*. [71].

Gene expression similarities between the brain and testis have prompted research groups to investigate the relationships between cognitive function and reproductive performance. Arden *et al*. [73] reported a positive correlation between intelligence and semen quality characteristics, including sperm concentration, count, and motility, in male humans. There is accumulating evidence suggesting that mutations in the same genes in both the brain and the testis could simultaneously cause dysfunctions of these tissues. The Huntington’s disease gene, huntingtin, is highly expressed in the brain and testis. The inactivation of the huntingtin gene in the forebrain and testis resulted in neuronal degeneration and sterility in the mouse [74]. Also, Kitamura *et al*. [75] showed that a mutation in the *ARX* gene, which is highly expressed in the brain and testis, caused abnormal forebrain and testis development in the mouse. Methylation changes in the sperm of old fathers have been found in genes involved in neuropsychiatric disorders and associated with schizophrenia and autism disorders in the offspring [76]. Taken together, these studies indicate that genes involved in the development of the central nervous system are susceptible to stochastic epigenetic variation that subsequent generations can inherit. Thus, the effects of methyl donors on sperm and neurological functions warrant further investigation.

### Correlation between DNA methylation and gene expression in TEI genes

Twelve TEI genes showed correlations between DNA methylation and expression levels (Supplementary Table S3). *CNTNAP2* is primarily involved in neuronal development and is associated with various disorders, including schizophrenia, Alzheimer’s disease, autism spectrum disorder, intellectual disability, dyslexia, and language impairment [77]. Also, *CNTNAP2* was found to be related to male infertility [78]. The protein encoded by *CNTNAP2* is shared between sperm and neural proteome, suggesting common functions in sperm and brain [71]. *IL1RAP* has been reported to have roles in immune response regulation [79], pathogenesis of Alzheimer’s disease [80], and autism spectrum disorders [81]. Interestingly, prenatal and early childhood infections have been implicated in autism such that many autism susceptibility genes are localized in the immune system and are related to immune/infection pathways [82]. As discussed in the previous section, *LPAR1* and *THOC1* were also associated with sperm-related traits and are expressed in the brain and testis. *AXDND1* is essential for spermatogenesis, and *Axdnd1* knock-out mice exhibited sterility caused by impaired spermiogenesis and abnormal nuclear shaping [83]. Thus, the correlation between methylation and expression levels in TEI genes escaping epigenetic reprogramming could shed new light on the biological significance of these genes and their roles in fetal programming. Furthermore, these data warrant further investigation of the link between brain and testis across generations.

### General considerations

In this study, we characterized and discussed the consequences of paternal diet on DNA methylation signatures in the offspring. We found that (i) many genomic regions resist global DNA demethylation and maintain their epimutations across generations; (ii) regions harboring TEs are susceptible to DNA methylation changes that are passed to subsequent generations; (iii) only two imprinted genes were found to resist epigenomic reprogramming, which could be explained by different reprogramming mechanisms regulating these genes [11]; (iv) some genomic regions escaping epigenetic reprogramming skip generations; and (v) genes escaping epigenetic reprogramming have functions related to fertility, growth, and neural development.

The data presented here provide a reference for the epigenetic status of genes potentially involved in maintaining and regulating fetal development during early life, a period expected to be particularly prone to epigenetic alterations induced by environmental and nutritional stressors. This is consistent with the Paternal Origins of Health and Disease paradigm, explaining how environmental challenges affect sperm methylation and can potentially affect the health of the offspring [84]. Also, the sperm DNA methylome facilitates mature gamete function, guides early embryogenesis, and influences later life [85]. Thus, sperm methylation marks could be potential biomarkers for environmentally induced epigenetic transgenerational inheritance of diseases [86].

The initial observation that imprinted genes are protected from global DNA methylation erasure during embryo development supports the idea that other genomic regions might escape epigenetic reprogramming and that these regions are TEI candidates [87]. For example, the *TRIM28* gene is required for epigenetic reprogramming, maintenance of DNA methylation during reprogramming, and genomic imprinting and epigenetic stability during the maternal-to-zygote transition [88]. Also, it has been shown that ZFP57 binds to the imprinted control regions and recruits DNA methyltransferases to protect imprinted genes from DNA methylation erasure in humans and mice [89, 90]. Indeed, in this study, we found many DMCs located in TEs, imprinted genes, nonimprinted genes, and other genomic regions that were altered by paternal diet and transmitted to subsequent generations.

It is well-accepted that the global erasure of DNA methylation in PGCs and pre-implantation embryos is a significant barrier to epigenetic inheritance in mammals [6]. However, the discovery in this and other studies of genomic regions escaping the two waves of epigenetic reprogramming supports the possibility of transgenerational epigenetic inheritance in mammals.

## Material and methods

### Experimental design

The experimental design is described in Ref. [39]. In brief, 10 male Polypay sheep twin pairs (20 rams in total) were randomly separated into two dietary treatments. A control diet was provided to one ram from each pair, and the other twin received the control diet plus 1.5 g rumen-protected methionine daily from weaning until puberty. Then, the rams were housed in individual pens with a group of untreated ewes for two breeding cycles to produce the F1 generation (*n* = 225 animals; 115 males and 110 females). Similarly, 10 F1 rams (5 F0 control offspring and 5 F0 treatment offspring) were individually housed with untreated ewes for two breeding cycles to produce F2 generation (*n* = 188 animals: 94 males and 94 females). Only a subset of the ewes used to generate the F1 generation were also used to generate the F2 generation. The average inbreeding coefficients for the F1 and F2 generations were 0.067 and 0.071, respectively, similar to the inbreeding coefficient of the entire flock (0.074). All F1 and F2 rams and ewes were fed a control diet throughout the trial.

### DNA methylation analysis of sperm

To investigate the transmission of altered epigenetic marks to the next generations, semen samples were collected from the F0, F1, and F2 generations, and DNA was extracted and subjected to whole-genome bisulfite sequencing (WGBS) analysis. Semen collection and DNA extraction were described in Refs [39] and [12]. We selected 40 samples for WGBS, including 10 F0 rams used to produce the F1 generation (five from the control group and their five co-twins from the methionine treatment group), 10 F1 samples (pooled), and 20 from the F2 generation (10 descendants from the F0 control group and 10 from the F0 treatment group) as described in [12]. All samples from the F0 and F2 generations were individually sequenced. From the F1 generation, 10 pooled samples were sequenced from 45 rams total, of which five pools contained samples from the offspring of the F0 control group and five pools composed of samples from the offspring of the F0 treatment group. Forty whole-genome bisulfite sequencing libraries were constructed and sequenced on the NovaSeq 6000 at the Roy J. Carver Biotechnology Center at the University of Illinois at Urbana–Champaign (UIUC). The shotgun genomic DNA libraries were prepared from 400 ng of DNA after sonication with a Covaris ME220 (Covaris, MA, USA) to an average fragment size of 400 bp with the Hyper Library Preparation Kit from Kapa Biosystems (Roche, Indianapolis, IN, USA), using methylated adaptors from Illumina. Bisulfite conversion was performed with the EZ-DNA Methylation Lightning kit (Zymo Research, Irvine, CA, USA). PCR amplification was carried out with the Kapa HiFi HotStart Uracil enzyme (Roche). The amplified libraries were quantitated with Qubit (Thermo Fisher, Waltham, MA, USA) and run on a fragment analyzer (Agilent Technologies, Santa Clar, CA, USA) to confirm the absence of free primers and primer dimers, ensuring the presence of DNA fragments within the anticipated size range. Libraries were pooled in equimolar concentration, and the pools were further quantitated by qPCR on a Bio-Rad CFX Connect Real-Time System (Bio-Rad Laboratories, Hercules, CA, USA). The pooled barcoded libraries were subsequently loaded on two NovaSeq S4 lanes for cluster formation and sequenced for 150 cycles from each side of the DNA fragments, generating 150 bp paired-end reads. Samples were demultiplexed, and quality checked using bcl2fastq Conversion Software (version 2.20, Illumina) and FastQC software (version 0.11.8, http://www.bioinformatics.babraham.ac.uk/projects/fastqc/), respectively. Reads were trimmed with TrimGalore (version 0.6.5, https://www.bioinformatics.babraham.ac.uk/projects/trim_galore/) using the options “--clip_R1 8 --clip_R2 8 --three_prime_clip_R1 10 --three_prime_clip_R2 10” to avoid poor qualities or biases. The trimmed data were aligned to the sheep reference genome (Oar_rambouillet_v1.0) using the “-X 1500”option, and deduplicated using the Bismark software with default parameters [91]. Further, cytosine methylation levels were called at a single-base resolution assessing cytosines in CG, CHH, or CHG contexts, where H represents A, C, or T [91]. Samples with at least 10 counts remained for downstream analysis. DMCs were identified using the “methylKit” R package [92] based on methylation difference >20% between the methionine treatment and control groups and with a FDR of 1%. The CG, CHH, or CHG contexts and each generation were analyzed separately, as described in Ref. [12].

### RNA sequencing of sperm

RNA sequencing was performed in the same 20 semen samples with WGBS from the F2 generation to identify genes affected by TEI DMCs. Prior to the RNA extraction, RNAlater-preserved samples (200 µl) were centrifuged for 4 min at 4000 rpm, supernatant was removed, and cells were suspended with 1 ml of somatic cell lysis buffer for 4 min on ice [93]. Samples were centrifuged for 4 min at 4000 rpm, and the lysis supernatant was removed. Later, total RNA was extracted using 1 ml of TRIzol Reagent (Life Technologies, Carlsbad, CA, USA), according to the manufacturer’s instructions, and DNase I treatment (Lucigen, Middleton, WI, USA) was applied. RNA sequencing for the 20 samples was performed at the Roy J. Carver Biotechnology Center, University of Illinois at UIUC, using the Illumina NovaSeq 6000 sequencing platform, generating 100-bp single-end reads. To generate and demultiplex the Fastq files, the bcl2fastq Conversion Software (version 2.20, Illumina) was used. A quality check of raw reads was performed using FastQC software for each sample separately. Trimmomatic [94] was then used to remove adapter sequences and low-quality reads and bases. Later, the alignment of the trimmed reads to the sheep reference genome (Oar_rambouillet_v1.0) was performed using STAR [95], including the “--quantMode GeneCounts” option to estimate gene counts. Expressed genes with at least 15 counts in more than 10 samples were considered for further analysis (16 098 in total). Gene count normalization was performed using the R package “edgeR” [96] based on the trimmed mean of M-values method.

The coefficient of linear correlation between the methylation levels (ratio of the intensities of methylated and unmethylated cytosines) of DMCs located within or in promoter regions [(10 kb from the transcription start site (TSS)] and the normalized expression values of the corresponding genes was calculated based on the Pearson correlation method [97]. Correlations with *P*  ≤ 0.10 and *r* ≥ 0.30 were considered significant [97].

### Identification of genomic regions escaping epigenetic reprogramming

TEI DMCs were defined as DMCs found across F0, F1, and F2 generations. Identified DMCs overlapping between F0 and F2 (F0–F2 DMCs) or F1 and F2 (F1–F2 DMCs) were also analyzed. Candidate genes escaping reprogramming (TEI genes) were considered those with TEI DMCs within the gene body or 10 kb upstream of a gene’s TSS. TEI genes were characterized as genes that harbor repeated elements (RE genes), along with imprinted genes, and genes that do no not harborrepeated elements (non-RE genes). Imprinted gene information was gathered from the Geneimprint database (http://www.geneimprint.com/). Repetitive element information was retrieved from the University of California, Santa Cruz database [98]. Functional enrichment analysis of genes harboring TEI, F0–F2, and F1–F2 DMCs was performed using gProfiler [99] based on Gene Ontology, adjusting for FDR of 5%.

## Supplementary Material

dvad010_Supp

## Data Availability

No new data were generated or analyzed in support of this research.
