# Supplementary material for: Characterization of genomic regions escaping epigenetic reprogramming in sheep
Source: Environ Epigenet. 2023 Dec 20;10(1):dvad010. doi: 10.1093/eep/dvad010 (PMC10944287; doi:10.1093/eep/dvad010)
Supplement: dvad010_Supp [file dvad010_supp.zip › suppl_data/Supplementary Table S3.docx]

**Supplementary Table 3.** Transgenerational epigenetic inheritance genes showing correlation between DNA methylation and gene expression

| Gene ID | Function | Reference | Correlation between DNA methylation and gene expression | P value |
| --- | --- | --- | --- | --- |
| YBX3 | Required for metazoan development and male fertility, as it is related to protamine biosynthesis of sperm. | (Snyder et al., 2015) | 0.43 | 0.058 |
|  | An intron region of this gene is associated with growth traits in chickens. | (Li et al., 2022) |  |  |
| STYK1 | Oncogene is expressed in the brain. It is associated with intracerebral hemorrhage, probably due to the capability of remodeling blood vessels in the brain. | (Yamada et al., 2017) | 0.414 | 0.070 |
| PCBD2 | Rare variants in this gene were associated with schizophrenia. | (González-Peñas et al., 2019) | 0.402 | 0.079 |
| DLG2 | Involved in hippocampal synaptic integration and plasticity, thus associated with schizophrenia, intellectual disability, and autism spectrum disorders | (Yoo et al., 2020) | 0.572 | 0.008 |
| AXDND1 | Required for spermatid differentiation; associated with male sterility | (Hiradate et al., 2022) | 0.432 | 0.057 |
| CNTNAP2 | Associated with autism spectrum disorders and epilepsy | (Peñagarikano et al., 2011; Poot, 2015; Fang et al., 2021) | -0.453 | 0.045 |
| IL1RAP | Involved in neuronal synapse and neuronal development; associated with Alzheimer's disease and schizophrenia | (Zettergren et al., 2019; Li et al., 2020; Cheng et al., 2022) | 0.390 | 0.089 |
| LPAR1 | Essential for maintaining the normal functions of the central nervous system; alterations in function or expression associated with neurodevelopmental and neuropsychiatric disorders and brain cancer | (Xiao et al., 2021) | 0.451 | 0.046 |
|  | Associated with semen quality in Holstein-Friesian bulls | (Kamiński et al., 2018) |  |  |
| THOC1 | Involved in presynaptic development and plays roles in dopamine neuron survival. Associated with herding behavior (fear) in dogs | (Maeder et al., 2018) | 0.381 | 0.097 |
|  | Required for testis and embryo development in mice | (Wang et al., 2009) |  |  |
| LOC105602588 |  |  | 0.436 | 0.055 |
| LOC105613000 (CD94-like) |  |  | 0.515 | 0.020 |
| LOC101116641 (CD94-like) |  |  | 0.449 | 0.047 |

**Supplementary Table 3 References**

Cheng P, Zhang R, Shan S, Yuan B, Chen J, Qiu Z and Du Y 2022. Novel IL1RAP mutation associated with schizophrenia interferes with neuronal growth and related NF-κB signal pathways. Neuroscience Letters 775, 136533.

Fang F, Ge M, Liu J, Zhang Z, Yu H, Zhu S, Xu L and Shao L 2021. Association between Genetic Variants in DUSP15, CNTNAP2, and PCDHA Genes and Risk of Childhood Autism Spectrum Disorder. Behavioural Neurology 2021, 1–6.

González-Peñas J, Costas J, Villamayor MJG and Xu B 2019. Enrichment of rare genetic variants in astrocyte gene enriched co-expression modules altered in postmortem brain samples of schizophrenia. Neurobiology of Disease 121, 305–314.

Hiradate Y, Harima R, Yanai R, Hara K, Nagasawa K, Osada M, Kobayashi T, Matsuyama M, Kanno S, Yasui A and Tanemura K 2022. Loss of *Axdnd1* causes sterility due to impaired spermatid differentiation in mice. Reproductive Medicine and Biology 21.

Kamiński S, Hering DM, Kordan W, Lecewicz M and Sazanov A 2018. Influence of splicing mutation within the lysophosphatidic acid receptor 1 gene (LPAR1) on semen quality in Holstein-Friesian bulls. Polish Journal of Veterinary Sciences 21, 419–421.

Li T, Qin P, Chen B, Niu X, Wang Y, Niu Y, Wei C, Hou D, Ma H, Han R, Li H, Liu X, Kang X and Li Z 2022. A novel 27-bp indel in the intron region of the *YBX3* gene is associated with growth traits in chickens. British Poultry Science 63, 590–596.

Li F, Zhang W, Wang M and Jia P 2020. IL1RAP regulated by PRPRD promotes gliomas progression via inducing neuronal synapse development and neuron differentiation in vitro. Pathology - Research and Practice 216, 153141.

Maeder CI, Kim J-I, Liang X, Kaganovsky K, Shen A, Li Q, Li Z, Wang S, Xu XZS, Li JB, Xiang YK, Ding JB and Shen K 2018. The THO Complex Coordinates Transcripts for Synapse Development and Dopamine Neuron Survival. Cell 174, 1436-1449.e20.

Peñagarikano O, Abrahams BS, Herman EI, Winden KD, Gdalyahu A, Dong H, Sonnenblick LI, Gruver R, Almajano J, Bragin A, Golshani P, Trachtenberg JT, Peles E and Geschwind DH 2011. Absence of CNTNAP2 Leads to Epilepsy, Neuronal Migration Abnormalities, and Core Autism-Related Deficits. Cell 147, 235–246.

Poot M 2015. Connecting the CNTNAP2 Networks with Neurodevelopmental Disorders. Molecular Syndromology 6, 7–22.

Snyder E, Soundararajan R, Sharma M, Dearth A, Smith B and Braun RE 2015. Compound Heterozygosity for Y Box Proteins Causes Sterility Due to Loss of Translational Repression. PLOS Genetics 11, e1005690.

Wang X, Chinnam M, Wang J, Wang Y, Zhang X, Marcon E, Moens P and Goodrich DW 2009. Thoc1 deficiency compromises gene expression necessary for normal testis development in the mouse. Molecular and Cellular Biology 29, 2794–2803.

Xiao D, Su X, Gao H, Li X and Qu Y 2021. The Roles of Lpar1 in Central Nervous System Disorders and Diseases. Frontiers in Neuroscience 15, 710473.

Yamada Y, Sakuma J, Takeuchi I, Yasukochi Y, Kato K, Oguri M, Fujimaki T, Horibe H, Muramatsu M, Sawabe M, Fujiwara Y, Taniguchi Y, Obuchi S, Kawai H, Shinkai S, Mori S, Arai T and Tanaka M 2017. Identification of six polymorphisms as novel susceptibility loci for ischemic or hemorrhagic stroke by exome-wide association studies. International Journal of Molecular Medicine 39, 1477–1491.

Yoo T, Kim S-G, Yang SH, Kim H, Kim E and Kim SY 2020. A DLG2 deficiency in mice leads to reduced sociability and increased repetitive behavior accompanied by aberrant synaptic transmission in the dorsal striatum. Molecular Autism 11, 19.

Zettergren A, Höglund K, Kern S, Thorvaldsson V, Johan Skoog M, Hansson O, Andreasen N, Bogdanovic N, Blennow K, Skoog I and Zetterberg H 2019. Association of IL1RAP-related genetic variation with cerebrospinal fluid concentration of Alzheimer-associated tau protein. Scientific Reports 9, 2460.
